# Identifying antinuclear antibody positive individuals at risk for developing systemic autoimmune disease: development and validation of a real-time risk model

**DOI:** 10.3389/fimmu.2024.1384229

**Published:** 2024-03-20

**Authors:** April Barnado, Ryan P. Moore, Henry J. Domenico, Sarah Green, Alex Camai, Ashley Suh, Bryan Han, Katherine Walker, Audrey Anderson, Lannawill Caruth, Anish Katta, Allison B. McCoy, Daniel W. Byrne

**Affiliations:** ^1^ Division of Rheumatology & Immunology, Department of Medicine, Vanderbilt University Medical Center, Nashville, TN, United States; ^2^ Department of Biomedical Informatics, Vanderbilt University Medical Center, Nashville, TN, United States; ^3^ Department of Biostatistics, Vanderbilt University Medical Center, Nashville, TN, United States

**Keywords:** antinuclear antibodies, electronic health record, risk model, autoimmune disease, rheumatology

## Abstract

**Objective:**

Positive antinuclear antibodies (ANAs) cause diagnostic dilemmas for clinicians. Currently, no tools exist to help clinicians interpret the significance of a positive ANA in individuals without diagnosed autoimmune diseases. We developed and validated a risk model to predict risk of developing autoimmune disease in positive ANA individuals.

**Methods:**

Using a de-identified electronic health record (EHR), we randomly chart reviewed 2,000 positive ANA individuals to determine if a systemic autoimmune disease was diagnosed by a rheumatologist. *A priori*, we considered demographics, billing codes for autoimmune disease-related symptoms, and laboratory values as variables for the risk model. We performed logistic regression and machine learning models using training and validation samples.

**Results:**

We assembled training (n = 1030) and validation (n = 449) sets. Positive ANA individuals who were younger, female, had a higher titer ANA, higher platelet count, disease-specific autoantibodies, and more billing codes related to symptoms of autoimmune diseases were all more likely to develop autoimmune diseases. The most important variables included having a disease-specific autoantibody, number of billing codes for autoimmune disease-related symptoms, and platelet count. In the logistic regression model, AUC was 0.83 (95% CI 0.79-0.86) in the training set and 0.75 (95% CI 0.68-0.81) in the validation set.

**Conclusion:**

We developed and validated a risk model that predicts risk for developing systemic autoimmune diseases and can be deployed easily within the EHR. The model can risk stratify positive ANA individuals to ensure high-risk individuals receive urgent rheumatology referrals while reassuring low-risk individuals and reducing unnecessary referrals.

## 1 Introduction

Positive antinuclear antibodies (ANAs) cause diagnostic dilemmas for clinicians across multiple specialties (1–3). Currently, no clinically available or validated tools exist to help clinicians determine the significance of a positive ANA. While a positive ANA serves as a diagnostic criterion for multiple autoimmune diseases, the test alone only has a 11% positive predictive value for systemic autoimmune disease (4). In US studies, rates of positive ANAs in the general population without autoimmune disease range from 14% to 27% (5, 6).

Frequent, inappropriate ordering of ANA testing has been recognized as a clinical problem by the American Board of Internal Medicine and the American College of Rheumatology in their “Choosing Wisely” campaign. Specifically, it is recommended to not order an ANA test unless specific symptoms for an autoimmune disease are present (7, 8). Up to 22% of all rheumatology referrals are for a positive ANA (1, 9). Only 11-20% of individuals with a positive ANA have an autoimmune disease diagnosed at referral (4, 10–13). Frequent ANA referrals in the setting of an international shortage of pediatric and adult rheumatologists (14–16) contribute to inefficient use of limited resources and lengthen wait times for rheumatology consultation (1, 9, 12).

Triage systems and electronic consultations have attempted to tackle the problem of frequent ANA referrals with limited success (12, 17–20). Risk models have been developed for systemic lupus erythematosus (SLE) (21, 22) but not for multiple systemic autoimmune diseases associated with a positive ANA. We aimed to develop and validate a robust risk model for use in the rheumatology clinic that uses readily available data in the electronic health record (EHR) to identify which individuals with a positive ANA are at high and low risk for developing systemic autoimmune disease.

## 2 Methods

### 2.1 Data source and patient selection

After receiving approval from the Vanderbilt University Medical Center (VUMC) IRB (#210189), we used the Synthetic Derivative, a de-identified version of the EHR that contains billing code and clinical data on over 3.6 million individuals spanning across three decades (23). Records from outside VUMC are not available.

We assembled all individuals within the Synthetic Derivative who had a positive ANA, defined as a titer ≥ 1:80 (**Supplementary Figure 1**). For ANA testing, the Hep-2 immunofluorescence assay was used for the entire study period (Appendix). We selected a random sample of 2,000 individuals with a positive ANA to perform chart review to assess for the model outcome and collect covariates. Model outcome was defined as developing a systemic autoimmune disease diagnosed by a rheumatologist, as EHR notes often lack systematic documentation of disease criteria (24). We performed chart review for development of systemic autoimmune disease from time of first positive ANA up to ten years later or individual’s last EHR interaction. We allowed up to ten years, as individuals with autoimmune diseases can face significant diagnostic delays (25). Systemic autoimmune diseases are listed in **Supplementary Table 1**. In addition to diseases classically associated with a positive ANA (i.e., SLE, Sjogren’s, systemic sclerosis, mixed connective tissue disease, and idiopathic inflammatory myopathies), we included other systemic autoimmune diseases such as rheumatoid arthritis (RA) and seronegative conditions (i.e., psoriatic arthritis, ankylosing spondylitis). Since the risk model will be used for triage to the rheumatology clinic, we aimed to include individuals with systemic autoimmune diseases who would be followed in that setting. While the ANA is not part of clinical criteria for these conditions, the ANA test is still frequently ordered in the evaluation of symptoms for these conditions (26). We excluded individuals with organ-specific autoimmune diseases such as autoimmune thyroiditis and autoimmune hepatitis, who would not be primarily managed by a rheumatologist. Individuals diagnosed outside of VUMC were included only if notes documented the individual was seen by an outside rheumatologist. For our primary analysis, we only analyzed individuals who were incident cases, defined as newly diagnosed with systemic autoimmune diseases at VUMC.

### 2.2 Model development

**Figure 1:**
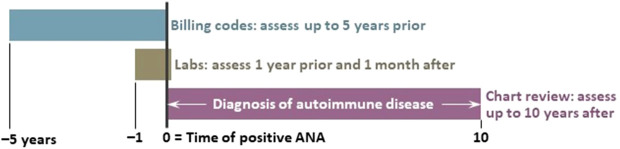
Timeline of model covariates. We assessed billing codes up to 5 years prior to the first positive antinuclear antibody (ANA) test. Laboratory values were assessed up to 1 year and 1 month after the ANA test. We conducted chart review for the model outcome of developing a systemic autoimmune disease diagnosed by a rheumatologist up to 10 years after the first positive ANA test.

Based on clinical relevance and published SLE risk models (21, 22), prespecified predictors included demographics, laboratory values, and billing codes up to the time of first positive ANA (**Supplementary Table 2**). Specifically, billing codes captured signs and symptoms for autoimmune diseases. A collection timeline for model covariates and outcome is detailed in **Figure 1**. Model outcome was developing a systemic autoimmune disease diagnosed by a rheumatologist within 10 years of first positive ANA (25).

Age was defined as age at first positive ANA documented at VUMC. The Synthetic Derivative defines race and ethnicity using a mixture of self-report and administrative entry with a fixed set of categories in accordance with NIH terminology. Studies have validated that these race and ethnicity assignments reflect self-report and genetic ancestry (27). For our primary analysis, race was initially excluded from the model as it was not significant in univariate analyses. Studies have shown that risk models that include race could potentially disadvantage high-risk groups from receiving appropriate care (28, 29). We performed a sensitivity analysis where race was included in the model, as studies demonstrate an increased risk of developing autoimmune disease in racial and ethnic underserved populations (1, 5).

We examined laboratory values one year prior to the date of the first positive ANA to allow for adequate data capture for individuals in the EHR and up to one month after to ensure capture of send-out studies such as the myositis antibody panel. We included autoantibodies associated with multiple autoimmune diseases (**Supplementary Table 3**). Autoantibodies were measured via enzyme-linked immunosorbent assays with manufacturer values to determine positivity (Appendix). We selected white blood cell count, platelet count, and serum creatinine as leukopenia, thrombocytopenia, and elevated serum creatinine have all been associated with autoimmune diseases (22, 30, 31). In SLE risk models (21, 22) and studies assessing presence of autoimmune diseases in positive ANA individuals (30, 31), leukopenia and thrombocytopenia were important predictors. Therefore, when examining multiple laboratory values for an individual, we selected the lowest white blood cell and platelet counts within the study period. For serum creatinine, we used the highest value within the study period to simulate how a rheumatologist might review lab trends. These values were treated as continuous variables. For missing laboratory values, we used median value imputation, as this method has been shown to be comparable to multiple imputation and is more feasible in real-time predictive models (32). We included ANA titer, as higher ANA titers are associated with risk of developing autoimmune disease (9, 30). Reporting of ANA titers are detailed in the Appendix. Briefly, ANA titer was dichotomized to 1:80 and ≥ 1:160 categories due to limited reporting of titers in some of the historical data. While different ANA patterns may have associations with different systemic autoimmune diseases (33), we did not include ANA pattern. ANA patterns are not reported in a standardized fashion at our institution according to the International Consensus on ANA patterns (33). Multiple or inconsistent patterns are often reported, particularly in the setting of changing technology over the study period. Further, as pattern is reported as a text variable, extraction from the EHR in real-time to input into the risk model would be challenging.

We used both ICD-9 and ICD-10-CM billing codes to capture signs and symptoms for systemic autoimmune diseases (**Supplementary Table 4**). These codes were significant in a UK SLE risk model (21) and were expanded upon to ensure capture of signs and symptoms for multiple autoimmune diseases in addition to SLE. Similar to the UK model, we searched for billing codes up to five years prior to the date of first positive ANA (21). In model development, we had an insufficient sample size to fit a model with a unique predictor for each billing code, so we created a single aggregated variable (**Supplementary Table 5**).

### 2.3 Statistical analysis

We derived separate training and validation sets using 2,000 positive ANA individuals. We estimated that 10-15% of our 2,000 positive ANA individuals would have an incident autoimmune disease (4, 10–13), leading to 200-300 cases for the training and validation sets combined. To prevent overfitting and applying the rule of 10-15 outcomes per one degree of freedom (34), we fit a logistic regression model with 13 degrees of freedom. Prespecified variables are shown in **Supplementary Table 2**. Total number of visits, white blood cell count, and serum creatinine were collinear with included model variables and were removed from the final model. We performed logistic regression using the following predictors: age at time of first positive ANA, sex, ANA titer, platelet count, and billing codes. Final model formula is in **Supplementary Figure 2**. We also performed machine learning methods including extreme gradient boosting (XGB) (35–37) and neural networks. Hyperparameters are in the Appendix. We assessed model performance in the training and validation sets using c-statistic, Brier score, and calibration curves.

### 2.4 Model validation

We conducted an internal validation of the logistic regression model using a bootstrap with 200 replications (38, 39). The bootstrap validation can test the stability of a model across different samples. In addition, a random selection of individuals, separate from the training set, was set aside as a “hold-out” for model validation (**Supplementary Figure 1**). Specifically, we estimated needing 100-200 incident autoimmune disease cases to avoid overfitting our model. To achieve this sample, we used 1384 individuals of which 1030 incident individuals were used for analysis, resulting in 152 incident cases. We then used the remainder of the original 2,000 set for a validation set with 616 individuals, of which 449 incident individuals were used for analysis, resulting in 74 incident cases.

### 2.5 Sensitivity analyses and deployment feasibility assessment

For our primary analysis, we excluded subjects with “unclear” autoimmune diagnoses. In a sensitivity analysis, we treated “unclear” subjects as not cases. We also included a sensitivity analysis where race was included with categories of White, Black, and Other. To account for longitudinal and censored data, we conducted a Cox proportional-hazard model using the same variables as the logistic regression model. Outcome was time from first positive ANA to either autoimmune disease diagnosis or last EHR follow-up (Appendix). We initially dichotomized ANA titer to 1:80 and ≥ 1:160 categories due to historical reporting in some of our data (Appendix). We then conducted a sensitivity analysis using more recent data (2017-2021) that incorporated multiple categories for the ANA titer (1:80, 1:160, 1:320, 1:640, 1:1280, and ≥ 1:2560). We also conducted sensitivity analyses where seronegative conditions were not counted as a case (Appendix).

We applied our logistic regression model to data extracted from our EHR-provided data warehouse (Epic Clarity) to assess feasibility of deploying the model in real-time. We calculated risk probabilities for systemic autoimmune disease for individuals with a positive ANA from 2017-2021. This time period captured the updated ANA titer reporting to the most current data available at time of analysis.

## 3 Results

### 3.1 Individual characteristics

Training (n = 1030) and validation (n = 449) sets are compared in **Table 1** with individuals having similar characteristics. In the training set, 15% (n = 152) of individuals with a positive ANA developed a systemic autoimmune disease. Individuals with systemic autoimmune diseases were younger (41.8 ± 21.5 vs. 47.9 ± 19.3 years, p = 0.003), more likely to be female (84% vs. 70%, p < 0.001), have a higher ANA titer (≥1:160 vs. 1:80) (90% vs. 79%, p = 0.002), lower serum creatinine (0.9 ± 0.6 vs. 1.2 ± 1.0 mg/dL, p < 0.001), higher platelet count (274 ± 113 vs. 229 ± 96 K/uL, p < 0.001), and a disease-specific autoantibody (51% vs. 9%, p < 0.001) (**Table 2**). No significant differences were found in race, ethnicity, or white blood cell count in individuals with vs. without systemic autoimmune diseases. Individuals with systemic autoimmune disease had a higher count of the nine billing code categories (scale 0 to 9) compared to individuals without disease (0.9 ± 0.9 vs. 0.6 ± 0.8, p < 0.001). Individuals with systemic autoimmune disease were more likely to have billing codes for arthritis (40% vs. 23%, p < 0.001) and Raynaud’s phenomenon (5% vs. 1%, p = 0.006) but not the other seven code categories.

**Table 1:** Characteristics of incident positive ANA individuals in training and validation sets.

Characteristics	Training set n = 1030	Validation set n = 449	*p* value^*^
**Autoimmune disease % (n)**	15% (152)	16% (74)	0.40
**Age at positive ANA**, years mean ± SD	47.0 ± 19.8	48.0 ± 20.3	0.44
**Race % (n)^†^** White	85% (807)	85% (355)	0.88
African American	12% (113)	12% (50)	
Asian	2% (16)	1% (5)	
Other	1% (11)	1% (5)	
**Ethnicity** ^†^ Hispanic	3% (32)	3% (11)	0.46
Not Hispanic or Latino/a	97% (889)	97% (397)	
**Sex**			
Female	72% (739)	74% (333)	0.34
**ANA titer‡**			
1:80	20% (202)	19% (87)	0.92
≥ 1:160	80% (828)	81% (362)	
**White blood cell count** ^†^ K/uL, Mean ± SD	6.9 ± 3.4	6.9 ± 2.9	0.88
**Platelet count** ^†^ K/uL, Mean ± SD	235 ± 100	233 ± 92	0.58
**Serum creatinine^†^** mg/dL, Mean ± SD	1.1 ± 0.9	1.2 ± 1.4	0.25
**Ever present autoantibody** ^§^ **% (n)**	15% (155)	15% (68)	0.96
**Total any billing codes** mean ± SD	30 ± 60	37 ± 71	0.27
**Count of specific billing codes** ^||^ mean ± SD	0.7 ± 0.8	0.8 ± 0.9	0.01
Alopecia % (n)	2% (21)	1% (6)	0.35
Arthritis	26% (264)	31% (140)	0.03
Fatigue	20% (207)	23% (104)	0.18
Interstitial Lung Disease	1% (14)	2% (11)	0.14
Pulmonary Hypertension	1% (11)	1% (6)	0.66
Rash	9% (97)	9% (42)	0.97
Raynaud’s	2% (19)	3% (12)	0.31
Serositis	4% (40)	5% (23)	0.28
Sicca	0.3% (3)	1% (5)	0.05

^*^Mann-Whitney U test for continuous variables and chi-square test for categorical variables. P values calculated with excluding missing observations.

†Race, ethnicity, and lab values have missing data with 81 (8%) for race, 109 (11%) for ethnicity, 201 (20%) for white blood cell count, 211 (20%) for platelet count, and 210 (20%) for serum creatine in the training set. In the validation set, 32 (7%) for race, 41 (9%) for ethnicity, 91 (20%) for white blood cell count, 95 (21%) for platelet count, and 100 (22%) for serum creatine.

‡For ANA titer, up until July 1, 2016, titers were reported as 1:40 (negative), 1:80, and ≥ 1:160. After this date, titers were then reported as 1:40 (negative), 1:80, 1:160, 1:320, 1:640, 1:1280, and 1:2560.

§Presence of other autoantibodies included rheumatoid factor, cyclic citrullinated peptide, SSA (Ro), SSB (La), scl-70, centromere, RNP, Smith, dsDNA, ANCA, Jo-1, or any antibody from the myositis antibody panel.

^||^See **Supplementary Table 4** for full list of ICD-9 and ICD-10-CM billing codes and **Supplementary Table 5** for details on scoring. For each individual, we counted if any billing code was ever present (1 for present, 0 for absent) for each of the nine categories (i.e., arthritis, fatigue) and then summed this up across the nine prespecified billing code categories for a maximum score of nine.

**Table 2:** Characteristics of positive ANA individuals with vs. without systemic autoimmune disease in the training set.

Characteristics	No systemic autoimmune disease n = 878	Systemic autoimmune disease n = 152	Proportion with systemic autoimmune disease*	*p* value^†^
**Age at positive ANA**, years, mean ± SD	47.9 ± 19.3	41.8 ± 21.5	··	0.003
**Race % (n)** ^‡^ White	85% (680)	85% (127)	16%	0.26
African American	12% (94)	13% (19)	17%	
Asian	2% (16)	0% (0)	0%	
Native American	0.1% (1)	1% (1)	50%	
Other	1% (10)	1% (1)	9%	
**Ethnicity** ^‡^ Hispanic	4% (30)	1% (2)	6%	0.13
Not Hispanic or Latino/a	96% (744)	99% (145)	16%	
**Sex** Female	70% (612)	84% (127)	17%	< 0.001
Male	30% (266)	16% (25)	9%	
**ANA titer** ^§^ 1:80	21% (186)	11% (16)	8%	0.002
≥ 1:160	79% (692)	90% (136)	16%	
White blood cell count^‡^				
K/uL, mean ± SD	6.9 ± 3.4	7.1 ± 3.2	··	0.49
**Platelet count** ^‡^ K/uL, mean ± SD	229 ± 96	274 ± 113	··	<0.001
**Serum creatinine** ^‡^ mg/dL, mean ± SD	1.2 ± 1.0	0.9 ± 0.6	··	<0.001
Ever present autoantibody^||^				
No	91% (800)	49% (75)	9%	<0.001
Yes	9% (78)	51% (77)	50%	
**Total any billing codes**, mean ± SD	32 ± 62	23 ± 43	··	0.02
**Count of specific billing codes**,^¶^ mean ± SD	0.6 ± 0.8	0.9 ± 0.9	··	< 0.001
Alopecia	2% (16)	3% (5)	24%	0.24
Arthritis	23% (203)	40% (61)	23%	< 0.001
Fatigue	19% (169)	25% (38)	18%	0.10
Interstitial Lung Disease	2% (13)	1% (1)	7%	0.42
Pulmonary Hypertension	1% (9)	1% (2)	18%	0.26
Rash	9% (81)	11% (16)	17%	0.61
Raynaud’s	1% (12)	5% (7)	37%	0.006
Serositis	4% (34)	4% (6)	15%	0.97
Sicca	0.3% (3)	0% (0)	0%	0.47

^*^Overall percentage of individuals with systemic autoimmune disease is 14.8%. P values calculated with excluding missing observations.

†Mann-Whitney U test for continuous variables and chi-square test for categorical variables.

‡Race, ethnicity, and lab values have missing data with 81 (8%) for race, 109 (11%) for ethnicity, 201 (20%) for white blood cell count, 211 (20%) for platelet count, and 210 (20%) for serum creatine.

§For ANA titer, up until July 1, 2016, titers were reported as 1:40 (negative), 1:80, and ≥ 1:160. After this date, titers were then reported as 1:40 (negative), 1:80, 1:160, 1:320, 1:640, 1:1280, and 1:2560.

^||^Presence of other autoantibodies included rheumatoid factor, cyclic citrullinated peptide, SSA (Ro), SSB (La), scl-70, centromere, RNP, Smith, dsDNA, ANCA, Jo-1, or any antibody from the myositis antibody panel.

^¶^See **Supplementary Table 4** for full list of ICD-9 and ICD-10-CM billing codes and **Supplementary Table 5** for details on scoring. For each individual, we counted if any billing code was ever present (1 for present, 0 for absent) for each of the nine categories (i.e., arthritis, fatigue) and then summed this up across the nine prespecified billing code categories for a maximum score of nine.

Of the 152 individuals with systemic autoimmune diseases, the most frequent diagnoses were SLE at 18% (n = 28) followed by other at 16% (n = 24), undifferentiated connective tissue disease at 16% (n = 24), and RA at 15% (n = 22) (**Supplementary Table 6**). Other consisted of psoriatic arthritis, unspecified inflammatory arthritis, and inflammatory bowel disease (**Supplementary Table 6**). Individuals with unclear diagnoses of systemic autoimmune disease (n = 66) were excluded from the primary analysis but are described in **Supplementary Table 7**. For individuals without systemic autoimmune diseases, when available alternative diagnoses were documented by rheumatologists, the most frequent diagnoses were fibromyalgia (n = 18), osteoarthritis (n = 11), and gout (n = 6) (**Supplementary Table 8**).

### 3.2 Model description and validation

**Figure 2:**
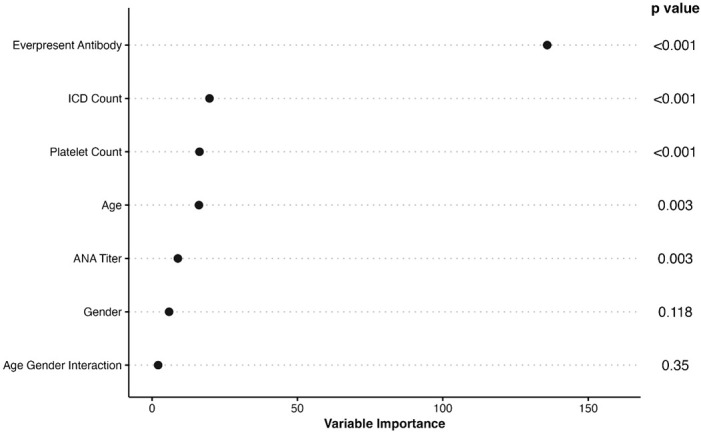
Importance of Variables in ANA Risk Model. The list of variables in the final ANA risk model are shown to the left with p values to the right. The x axis shows variable importance using a Wald statistic. Ever-present antibody refers to having a disease-specific autoantibody such as a rheumatoid factor or dsDNA. ICD count refers to billing code category count that ranges from 0 to 9.

**Figure 3:**
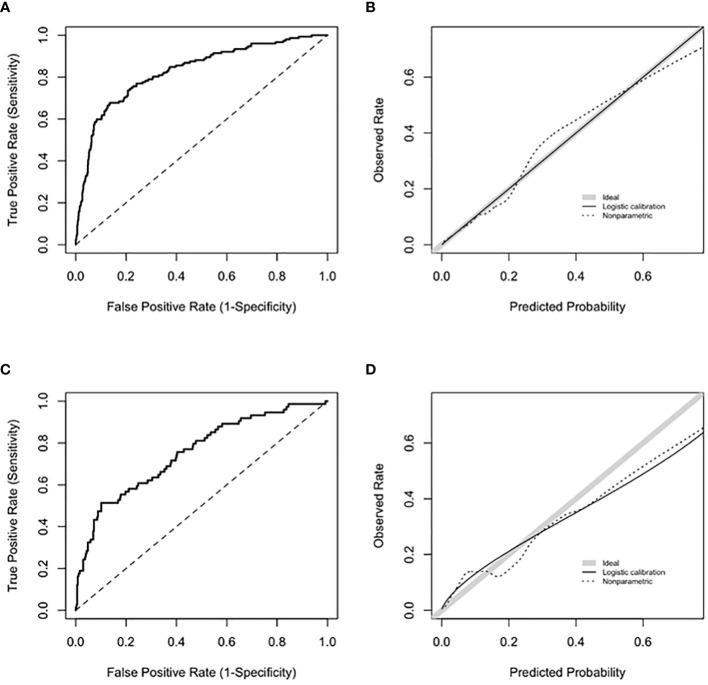
Model performance for training and validation sets. **(A)** shows ROC for the training set with an AUC 0.83 (95% CI 0.79-0.86). **(B)** shows calibration curve with a slope of 1 and intercept of 0 for the training set. Slopes that approach 1, as shown by the shaded grey line, demonstrate ideal calibration, agreement between predicted risk for systemic autoimmune disease and observed rate. **(C)** shows ROC for the validation set with an AUC 0.75 (95% CI 0.68-0.81). **(D)** shows calibration curve for the validation set. Calibration slope was equal to 0.71 and intercept was equal to 0.08.

The final model included age at first positive ANA, sex, ANA titer, presence of another autoantibody, platelet count, and billing code category count. Age was fit with a three-knot restricted cubic spline and interacted with sex and was prespecified based on prior literature (21). Our data demonstrated a higher probability of systemic autoimmune disease in female vs. male individuals at younger ages but a similar probability at older ages (**Supplementary Figure 3**). The most important variables in the model were presence of another autoantibody (i.e., dsDNA), billing code category count, and platelet count (**Figure 2**). Model AUC was 0.83 (95% CI 0.79-0.86) (**Figure 3A**) with a Brier score of 0.10 and calibration shown in **Figure 3B**. XGBoost resulted in an AUC of 0.94 (95% CI 0.91-0.95) and neural networks with an AUC of 0.83 (95% CI 0.79-0.87).

Based on the internal bootstrap validation, the logistic regression model was stable and robust (Appendix). For the validation set (n = 449), 16% of individuals had systemic autoimmune disease (**Supplementary Table 9**). For the logistic regression model, AUC was 0.75 (95% CI 0.68-0.81) (**Figure 3C**) with a Brier score of 0.12 with calibration shown in **Figure 3D**. XGBoost resulted in an AUC of 0.72 (95% CI 0.65-0.78) and neural networks with an AUC of 0.74 (95% CI 0.68-0.81).

### 3.3 Sensitivity analyses

Race was included in the model with categories of White, Black, and Other resulting in an AUC of 0.83 (95% CI 0.79-0.87). When individuals of unclear case status for systemic autoimmune disease were counted as non-cases, model AUC was 0.80 (95% CI 0.76-0.83). When these unclear individuals were counted as cases, model AUC was 0.74 (95% CI 0.71-0.77). The distribution of model risk scores for these unclear individuals most closely matched individuals who were not cases (**Supplementary Figure 4**). For the Cox model with the outcome time to autoimmune diagnosis, model predictors behaved similarly to the logistic regression model (**Supplementary Figure 5**).

To reflect more updated ANA titer reporting, we used a cohort of individuals with a positive ANA from 2017 to 2021 (n = 584) (Appendix) to perform additional sensitivity analyses. For the 2017-2021 cohort, there was a significant difference in the distribution of ANA titers between cases and non-cases (p < 0.001). Of the cases, 40% had an ANA titer greater than 1:640, while 18% of non-cases had a titer greater than 1:640 (**Supplementary Table 10**). In this cohort, using a dichotomized ANA titer (1:80 vs. ≥1:160), model AUC was 0.85 (95% CI 0.81 – 0.90). For the model with full ANA titer reporting (i.e., 1:80, 1:160, 1:320, 1:640, 1:1280, ≥ 1:2560), model AUC was 0.89 (95% CI 0.84 – 0.92). Lastly, we assessed if a higher ANA titer cutoff would impact model performance using the above 2017-2021 cohort. We fit a model using an ANA cutoff at 1:160, which had an AUC of 0.83 (95% CI 0.78-0.87), identical to the performance of the model using the original ANA cutoff at 1:80 (AUC of 0.83 (95% CI 0.78-0.87)).

For using an alternative case definition for systemic autoimmune disease that did not count seronegative conditions (i.e., psoriatic arthritis, ankylosing spondylitis) as cases, model AUC was 0.86 (95% CI 0.83-0.89).

### 3.4 Distribution of risk scores by type of autoimmune disease

We examined the distribution of model risk scores by type of autoimmune disease (**Supplementary Figure 6**). Individuals with SLE had the highest risk scores with a median of 0.481 and IQR of 0.312-0.685 followed by RA with 0.423 (0.144-0.582). Individuals labeled as other, with predominantly seronegative conditions, had the lowest median risk score of 0.107 (0.061-0.269). Seronegative conditions included psoriatic arthritis, and inflammatory bowel disease. Individuals with seropositive diseases had a higher median risk score compared to individuals with seronegative diseases (0.385 vs. 0.107, difference in medians = 0.278, 95% CI 0.195 – 0.332, p < 0.001).

### 3.5 Deployment feasibility

**Figure 4:**
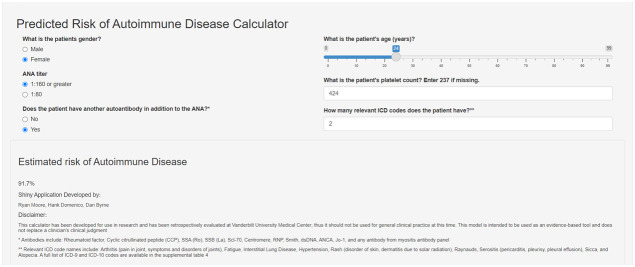
Screenshot of Shiny app for risk model for systemic autoimmune disease. The screenshot shows the risk model covariates used to estimate risk for systemic autoimmune disease. This app demonstrates how the risk score is calculated and is not intended for clinical practice. The Shiny app can be accessed at the following link: https://cqs.app.vumc.org/shiny/AutoimmuneDiseasePrediction/.

We assessed the feasibility of implementing the logistic regression risk model in our Epic EHR using data for all individuals with a positive ANA from 2017-2021 (n = 22,234). We observed a similar distribution of risk scores in Epic compared to our training set that used a de-identified EHR database (Synthetic Derivative) (**Supplementary Figure 7**). A demonstration of how the risk model works can be accessed at https://cqs.app.vumc.org/shiny/AutoimmuneDiseasePrediction/ (**Figure 4**). A disclaimer is included that the application is not intended for clinical practice.

## 4 Discussion

We developed and validated a risk model that predicts risk for developing systemic autoimmune disease in individuals with a positive ANA. The model is important because it utilizes readily available clinical data in the EHR, can be deployed easily within clinical practice, and helps risk stratify individuals with a positive ANA, a source of frequent rheumatology referrals. Our risk model identifies high-risk individuals, who are most likely to develop a systemic autoimmune disease, to ensure they are seen urgently for prompt diagnosis and treatment. Our risk model also identifies low-risk individuals who could be reassured, reducing unnecessary rheumatology referrals.

To our best knowledge, a risk model that focuses on individuals with a positive ANA and predicts risk for multiple systemic autoimmune diseases does not currently exist. One SLE risk model used UK EHR data (21) but did not focus on positive ANA individuals or examine risk for other autoimmune diseases. In this model, billing codes such as arthritis, rash, sicca, and fatigue were most significantly associated with risk of developing SLE along with female sex, younger age, and a higher number of clinic visits. We found similar results in our model and used similar billing codes but expanded our codes to identify not just SLE but also other systemic autoimmune diseases. Similar to the UK SLE model, we used a non-linear age and an age-sex interaction term. Despite its strengths, the UK SLE model had limited performance with a positive predictive value of 7-9%, a sensitivity of 24-34%, and an AUC of 0.75. Further, this model was not deployed in the EHR. Our model attained a higher AUC of 0.83 and can be easily deployed in real-time in the EHR.

Another SLE risk model from a Greek center (22) used random forests and Lasso-LR models. Not surprisingly, clinical items from the ACR SLE classification criteria accurately identified SLE cases with a high model AUC. While this study had a relatively large sample and a validation set, the model was developed using rheumatology clinic individuals and not in a general practice setting where there is often diagnostic dilemma. This model would be challenging to deploy in the EHR as it relies on SLE diagnostic criteria that may not be documented systematically, even in rheumatology notes (24).

The most important variable in our model was having another autoantibody in addition to the positive ANA, which is more specific for autoimmune diseases (1–3). Individuals with disease-specific autoantibodies may have a higher pretest probability for autoimmune disease by simply having these tests ordered. We tried to mitigate this bias by only including incident positive ANA individuals without established diagnoses of systemic autoimmune disease. Further, our institution conducts reflex testing where disease-specific autoantibodies are sent if an ANA is positive. Disease-specific autoantibodies may not be available fully in real-time at centers that do not perform reflex testing with a positive ANA, which may impact the performance of the model. The next most important variable was count of the nine prespecified billing code categories. *A priori*, we selected billing codes that captured signs and symptoms for autoimmune diseases and were significant in the UK SLE risk model (21). As expected, a higher count of these billing codes was predictive for systemic autoimmune disease. While billing codes may not always adequately capture an individual’s symptoms, ICD billing codes allow for automation of the risk model in real-time and allow for portability of the model to other EHRs and databases that use common data models. Platelet count was also an important variable in our model. We originally hypothesized that a lower platelet count would be associated with systemic autoimmune disease. Prior SLE risk models identified thrombocytopenia as an important model predictor (21, 22), and other studies demonstrated an association of thrombocytopenia with autoimmune disease in positive ANA individuals (30, 31). Instead, we found a higher value of an individual’s lowest platelet count was associated with systemic autoimmune disease. Higher platelet counts have been observed in individuals with RA and correlate with increased disease activity (40) and may also signal inflammation (41). *A priori*, we elected to not include inflammatory markers such as sedimentation rate (ESR) and C-reactive protein (CRP), as we had significant missingness of these values in the EHR. Further, these markers are nonspecific and can fluctuate widely in an individual (42–44). Elevations in these markers can be unrelated to an underlying systemic autoimmune disease, for example, in the setting of infection and malignancy (42–45).

*A priori*, we included race and ethnicity in our risk model. African American and Hispanic individuals have higher frequencies of positive ANAs compared to White individuals and are at higher risk of developing autoimmune disease, particularly SLE (1, 5). In univariate analysis, neither race nor ethnicity were significantly associated with systemic autoimmune disease, so race and ethnicity were not initially included. Studies have shown that risk models that include race could potentially disadvantage high-risk groups from receiving appropriate care (28, 29). For our model, this could include Black individuals. In a sensitivity analysis, we included race and found a similar model AUC of 0.83.

Our logistic regression model demonstrated robustness in both an internal bootstrap validation and a separate validation set. A successful bootstrap validation demonstrates the model can hold up when it encounters different samples. With predicting a clinically complex outcome where no current tools or risk models exist, our model validation demonstrated an improvement over usual care. To assess alternative approaches, we developed models using XGBoost and neural networks. XGBoost had a higher apparent AUC compared to the training set logistic regression model, likely due to overfitting, but did not hold up in validation. Neural networks performed similarly to the logistic regression model but with added complexity that would limit interpretability and deployment in the EHR.

While we developed, validated, and deployed a robust risk model to predict risk of systemic autoimmune disease in positive ANA individuals, our study has limitations. Our model was developed at a single academic medical center with more complex patients being evaluated, so may not generalize to other practice settings. Further, our study population was predominantly White, so it may not generalize to individuals with different race and ethnicity backgrounds and in other geographic areas. Our data encompasses an almost 30-year study period that included changes in ANA titer reporting. As a result, our primary analysis for the risk model included dichotomized reporting of the ANA titer to capture historical data. Sensitivity analyses using a more recent cohort of positive ANA individuals using both the dichotomized and full reporting of the ANA titer had similar model AUCs with overlapping confidence intervals. For future versions of the risk model, full reporting of the ANA titer can be used. We purposely defined systemic autoimmune disease based on a rheumatologist’s diagnosis instead of classification criteria, as classification criteria are not systematically documented in clinical notes (24). Case definition by a rheumatologist could contribute to heterogeneity of cases (i.e. calling an individual with mild SLE and SLE nephritis both SLE).

Interestingly, our model did not perform as well in individuals with seronegative conditions not typified by autoantibodies, as presence of these autoantibodies was the strongest predictor in our model. This limitation should be considered when interpreting risk scores. Seronegative conditions encompass overlapping diseases including plaque psoriasis, psoriatic arthritis, and inflammatory bowel diseases. These conditions have different HLA-based risk alleles, disease mechanisms, and disease presentations compared to seropositive conditions (46). While these seronegative conditions are not classically associated with a positive ANA, individuals with these conditions can have higher rates of ANA positivity compared to the general population (47–49) and often have an ANA test ordered as part of their clinical evaluation (26). In a sensitivity analysis, not counting the individuals with seronegative conditions as cases did not greatly impact the performance of the model.

Our model achieved a robust AUC of 0.83, but it does not discriminate perfectly between individuals with and without systemic autoimmune diseases. We found this AUC to be an improvement over usual care, where no current risk models exist to help risk stratify positive ANA individuals. The risk model was not designed to diagnose systemic autoimmune disease but to serve as a tool to identify positive ANA individuals who are at risk of developing systemic autoimmune disease within the next 10 years. The risk model can complement the clinician’s judgment as well as the patient history and physical exam. The risk model could also assist the ordering physician in identifying individuals at lower risk that may not need rheumatology referral. This reassurance may reduce unnecessary referrals and expenses to the healthcare system. We purposefully created a continuous risk score, which is more rigorous than commonly used dichotomous or “cut-off” scores. Without a “cut-off score,” we cannot currently estimate a positive predictive value. We are currently conducting a prospective validation of the risk model in real-time in the EHR to inform which individuals are low vs. high risk. While we created an application to demonstrate how the model incorporates variables and calculates a risk score, this application is not intended to be used in clinical practice yet or identify individuals as low vs. high risk.

In summary, we developed, validated, and deployed a risk model to identify which positive ANA individuals will develop systemic autoimmune disease. This risk model can be automated and deployed in real-time with no input needed from a clinician. In the setting of an international shortage of rheumatologists (14–16), a risk-stratifying tool for positive ANA individuals is critical. For future directions, we are assessing our risk model in real-time in the EHR prospectively and its impact on time to diagnosis and treatment for autoimmune diseases. Pending prospective validation, we envision our risk model would predict risk of autoimmune diseases within 10 years of a positive ANA similar to the FRAX that predicts 10-year fracture risk (50) or the ASCVD risk algorithm that predicts 10-year cardiovascular event risk (51). Risk scores from our model could then directly inform management of individuals with positive ANAs. High-risk individuals could be seen urgently by rheumatologists to ensure prompt diagnosis and treatment, and low-risk individuals could be reassured, reducing unnecessary rheumatology referrals.

## Data availability statement

Raw data and R code used in analyses will be available by the authors, without undue reservation.

## Ethics statement

The studies involving humans were approved by Vanderbilt University Medical Center. The studies were conducted in accordance with the local legislation and institutional requirements. Written informed consent for participation was not required from the participants or the participants’ legal guardians/next of kin in accordance with the national legislation and institutional requirements.

## Author contributions

AB: Conceptualization, Data curation, Formal analysis, Funding acquisition, Investigation, Methodology, Project administration, Resources, Software, Supervision, Validation, Visualization, Writing – original draft, Writing – review & editing. RM: Conceptualization, Data curation, Formal analysis, Methodology, Software, Validation, Visualization, Writing – original draft, Writing – review & editing. HD: Conceptualization, Formal analysis, Investigation, Methodology, Software, Supervision, Validation, Visualization, Writing – original draft, Writing – review & editing. SG: Data curation, Investigation, Project administration, Supervision, Writing – original draft, Writing – review & editing. AC: Data curation, Investigation, Writing – original draft, Writing – review & editing. AS: Data curation, Investigation, Project administration, Supervision, Writing – original draft, Writing – review & editing. BH: Data curation, Investigation, Writing – original draft, Writing – review & editing. KW: Data curation, Investigation, Writing – original draft, Writing – review & editing. AA: Data curation, Investigation, Writing – original draft, Writing – review & editing. LC: Data curation, Investigation, Writing – original draft, Writing – review & editing. AK: Data curation, Investigation, Writing – original draft, Writing – review & editing. AM: Investigation, Methodology, Project administration, Resources, Software, Writing – original draft, Writing – review & editing. DB: Conceptualization, Formal analysis, Investigation, Methodology, Software, Validation, Visualization, Writing – original draft, Writing – review & editing.
