# Supplementary material for: Identifying antinuclear antibody positive individuals at risk for developing systemic autoimmune disease: development and validation of a real-time risk model
Source: Front Immunol. 2024 Mar 20;15:1384229. doi: 10.3389/fimmu.2024.1384229 (PMC10987951; doi:10.3389/fimmu.2024.1384229)
Supplement: Supplementary file 1 [file DataSheet_1.docx]

Supplementary Material

**
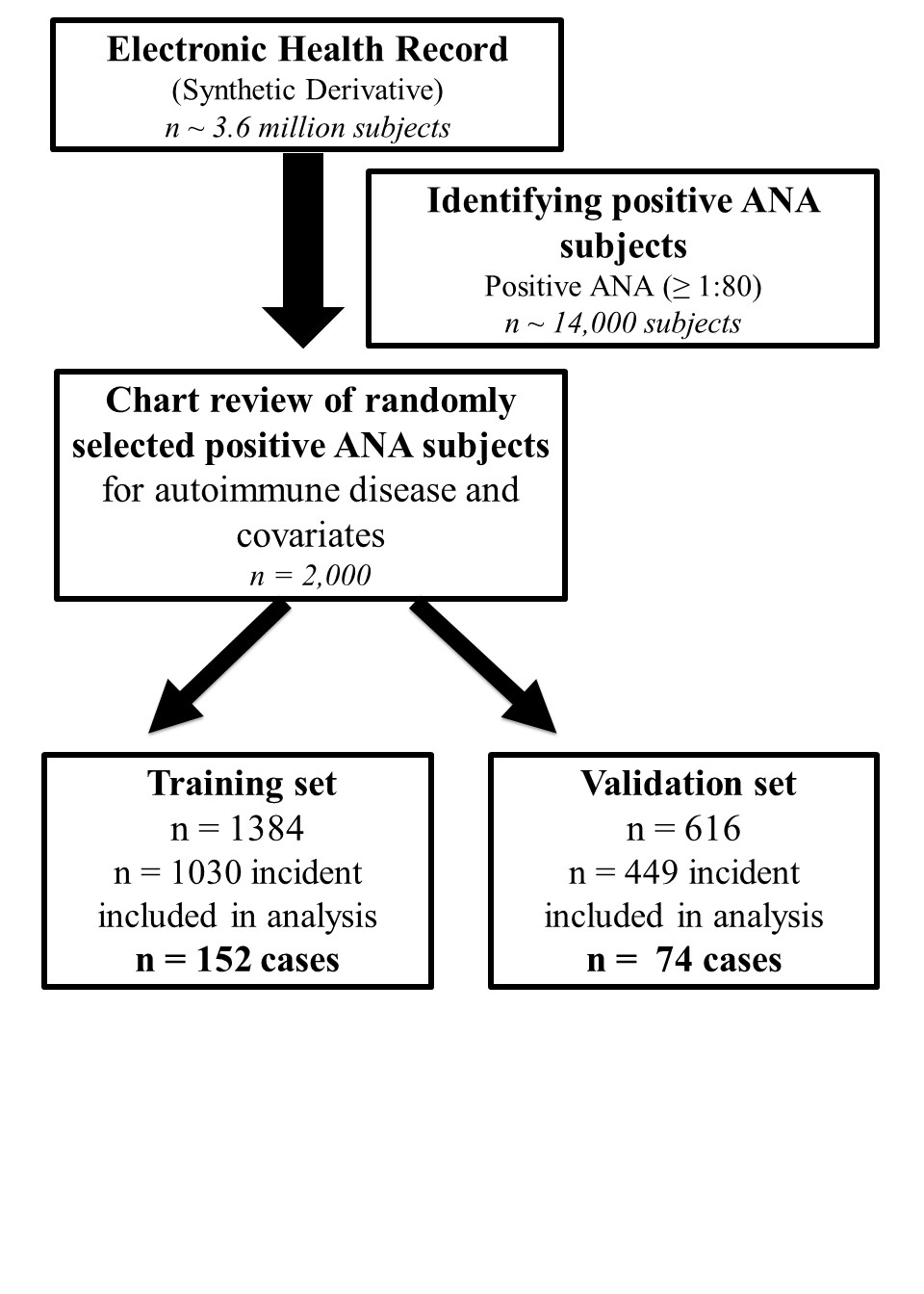
**

**Supplemental Figure 1. Flow chart of training and validation sets.** We used a de-identified version of the electronic health record (EHR) called the Synthetic Derivative. We identified individuals with a positive antinuclear antibody (ANA), defined as a titer ≥ 1:80. We then randomly selected 2,000 of these individuals for chart review to determine if diagnosed with a systemic autoimmune disease by a rheumatologist. We selected a training set aimed at identifying approximately 100-200 autoimmune disease cases for our needed sample size. We then used the remaining of the 2,000 individuals for our validation set.

**Supplemental Table 1. List of Systemic Autoimmune Diseases**

| **Systemic Autoimmune Diseases** |
| --- |
| Systemic lupus erythematosus (SLE) |
| Sjogren’s syndrome |
| Systemic sclerosis or scleroderma |
| Mixed connective tissue disease |
| Rheumatoid arthritis (RA) |
| Idiopathic inflammatory myopathies (i.e. Dermatomyositis, Polymyositis) |
| Undifferentiated connective tissue disease (UCTD) |
| Juvenile idiopathic arthritis (JIA) |
| Antineutrophil cytoplasmic antibodies (ANCA)-associated vasculitis |
|  |
| **Other^*^** |
| Plaque psoriasis |
| Psoriatic arthritis |
| Ankylosing spondylitis |
| Ulcerative colitis |
| Crohn’s disease |
| Undifferentiated inflammatory arthritis |
| Sarcoidosis |
| Behcet’s disease |

^*^List of systemic autoimmune diseases that are typified by the absence of disease-specific autoantibodies, i.e., rheumatoid factor or dsDNA, and often referred to and grouped as seronegative autoimmune diseases.

**Supplemental Table 2. Prespecified Variables for Autoimmune Disease Risk Model.**

| **Demographics**  Age at time of positive ANA |
| --- |
| Sex |
| Race |
| Ethnicity |
| **Billing code categories** |
| Arthritis |
| Rash |
| Alopecia |
| Raynaud’s |
| Sicca |
| Fatigue |
| Serositis |
| Interstitial lung disease or pulmonary hypertension |
| **Laboratory data** |
| ANA titer (1:80 or ≥ 1:160) |
| Lowest white blood cell count |
| Lowest platelet count |
| Highest serum creatinine |
| Ever present rheumatic disease-associated autoantibody |

**Supplemental Table 3. List of autoantibodies.**

| **Autoantibodies** |
| --- |
| Rheumatoid factor |
| Cyclic citrullinated peptide (CCP) |
| SSA (Ro) |
| SSB (La) |
| Scl-70 |
| Centromere |
| RNP |
| Smith |
| dsDNA |
| ANCA |
| Jo-1 |
| Any antibody from myositis antibody panel* |

*****Myositis antibody panel is a send-out study to ARUP Laboratories. Antibodies in the panel include Ribonucleic protein (RNP) U1, SSA 52, Jo-1, PM/Scl 100, Mi-2, PL-7, PL-12, TIF1-gamma, EJ, Ku, SRP, OJ, SSA 60, Fibrillarin (U3 RNP), SAE1, NXP2, and MDA5.

**Supplemental Table 4. Billing Codes Used for ANA Risk Model.**

| **Name of Billing Code** | **ICD-9 Billing Codes** | **ICD-10-CM Billing Codes** |
| --- | --- | --- |
| Arthritis (pain in joint, symptoms and disorders of joints) | 719.4*, 719.0*, 719.5*, 719.6*, 719.79, 719.8*, 719.9* | M25.5*, M79.64*, M12.0*, M25.4*, M25.6*, M25.8*, M25.9 |
| Rash (disorder of skin, dermatitis due to solar radiation) | 782.1, 709, 709.8, 709.9, V13.3, 692.70, 692.72, 692.74, 692.79, 692.82 | R21, L44.8, L44.9, L45, L98.8, L98.9, L99, Z87.2, L56.8, L56.9, L59.8, L59.9 |
| Alopecia | 704.0, 704.00, 704.8, 704.9 | L65.0, L65.8, L65.9, L66.8, L66.9 |
| Raynaud’s | 443.0 | I73.00, I73.01 |
| Sicca | 370.33 | H16.22* |
| Fatigue | 780.7, 780.71, 780.79 | R53, R53.0, R53.82, R53.83 |
| Serositis (pericarditis, pleurisy, pleural effusion) | 420, 420.0, 420.9, 420.90, 420.91, 420.99, 423.8, 423.9, 511, 511.0, 511.8, 511.89, 511.9 | I30.0, I30.8, I30.9, I31.8, I31.9, I32, M32.12, J90, J91.8, R09.1 |
| Interstitial lung disease | 516.3, 516.30, 516.31, 516.32, 516.33, 516.34, 516.35, 516.36, 516.37 | J84.1, J84.10, J84.11, J84.111, J84.112, J84.113, J84.114, J84.115, J84.116, J84.117, J84.17, J84.2, J84.8, J84.89, J84.9 |
| Pulmonary hypertension | 416.0 | I27.0, I27.2, I27.2, I27.22, I27.23, I27.24, I27.29, I27.83 |

**Supplemental Table 5. Example of Scoring for Billing Codes.**

| **Billing Code Categories*** | **Individual A** | **Individual B** |
| --- | --- | --- |
| Arthritis | 1 | 0 |
| Rash | 1 | 0 |
| Alopecia | 0 | 0 |
| Raynaud’s | 0 | 1 |
| Sicca | 0 | 0 |
| Fatigue | 1 | 0 |
| Serositis | 0 | 0 |
| Interstitial lung disease | 0 | 1 |
| Pulmonary hypertension | 0 | 0 |
| Total score | 3 | 2 |

*****If an individual has any of the ICD-9 or ICD-10-CM codes ever present within each of the nine categories listed above, the individual is assigned a score of 1. If the codes are never present, then the individual is assigned a score of 0. The score is then summed and can range from 0 to 9.

**
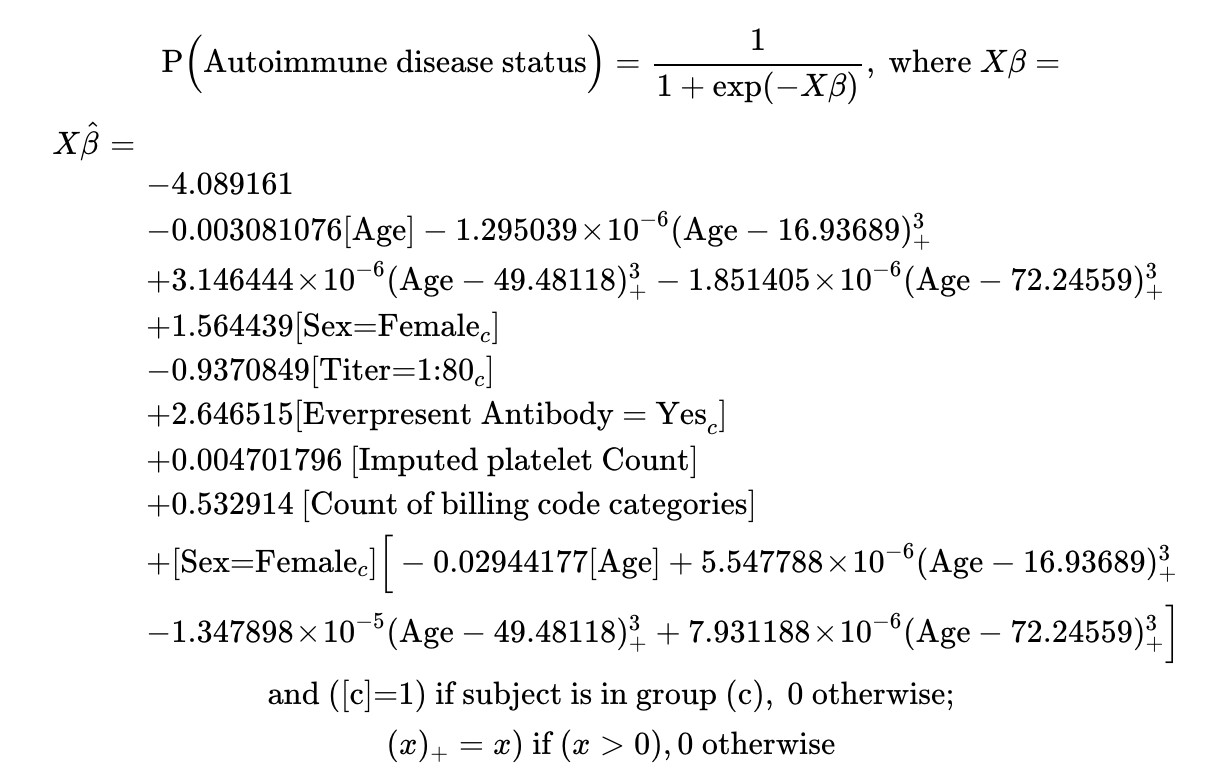
**

**Supplemental Figure 2. Formula for systemic autoimmune disease risk model.** If platelet count was missing, the median value 237 was imputed. Count of billing code categories refers to the scoring of 9 billing code categories (Arthritis, Fatigue, Interstitial lung disease, Hypertension, Rash, Raynaud’s, Serositis, Sicca, and Alopecia). If an individual has any of the ICD-9 or ICD-10-CM codes ever present within each of the nine categories listed above, the individual is assigned a score of 1. If the codes are never present, then the individual is assigned a score of 0. The final variable is the sum of the 9 billing code categories with a score ranging from 0 to 9.

**Supplemental Table 6. Autoimmune Disease Diagnoses in Cases in Training Set.**

| **Disease Type** | **n (%)**  **n = 152** |
| --- | --- |
| Systemic Lupus Erythematosus | 28 (18%) |
| Other* | 24 (16%) |
| Undifferentiated Connective Tissue Disease | 24 (16%) |
| Rheumatoid Arthritis | 22 (15%) |
| Juvenile Idiopathic Arthritis | 15 (10%) |
| Sjogren’s | 14 (9%) |
| Dermatomyositis/Polymyositis | 12 (8%) |
| Systemic sclerosis | 8 (5%) |
| Mixed connective tissue disease | 5 (3%) |

*Other includes psoriatic arthritis/plaque psoriasis (n = 8), inflammatory arthritis (n = 8), inflammatory bowel disease (n = 3), Adult-onset Still’s disease (n = 1), Ankylosing spondylitis (n = 1), Henoch Schoenlein Purpura (n = 1), Polymyalgia rheumatica (n = 1), and sarcoidosis (n = 1).

**Supplemental Table 7. Characteristics of positive ANA individuals with vs. without systemic autoimmune disease and individuals with unclear diagnoses in the training set.**

| **Characteristics** | **No systemic autoimmune disease**  **n = 878** | **Systemic autoimmune disease**  **n = 152** | **Individuals with unclear diagnoses***  **n = 66** |
| --- | --- | --- | --- |
| **Age at positive ANA**, years, mean ± SD | 47.9 ± 19.3 | 41.8 ± 21.5 | 51.3 ± 14.6 |
| **Race % (n)**^‡^  White | 85% (680) | 85% (127) | 84% (50) |
| African American | 12% (94) | 13% (19) | 12% (7) |
| Asian | 2% (16) | 0% (0) | 2% (1) |
| Native American | 0.1% (1) | 1% (1) | 0% |
| Other | 1% (10) | 1% (1) | 2% (1) |
| **Ethnicity**^‡^  Hispanic | 4% (30) | 1% (2) | 2% (1) |
| Not Hispanic or Latino | 96% (744) | 99% (145) | 98% (54) |
| **Gender**  Female | 70% (612) | 84% (127) | 79% (52) |
| Male | 30% (266) | 16% (25) | 21% (14) |
| **ANA titer**^§^  1:80 | 21% (186) | 11% (16) | 20% (13) |
| ≥ 1:160 | 79% (692) | 90% (136) | 80% (53) |
| **White blood cell count**^‡^  K/uL, mean ± SD | 6.9 ± 3.4 | 7.1 ± 3.2 | 7.4 ± 3.6 |
| **Platelet count**^‡^  K/uL, mean ± SD | 229 ± 96 | 274 ± 113 | 247 ± 93 |
| **Serum creatinine**^‡^  mg/dL, mean ± SD | 1.2 ± 1.0 | 0.9 ± 0.6 | 1.2 ± 1.1 |
| **Ever present autoantibody**^\|\|^  No | 91% (800) | 49% (75) | 86% (57) |
| Yes | 9% (78) | 51% (77) | 14% (9) |
| **Total any billing codes,** mean ± SD | 32 ± 62 | 23 ± 43 | 33 ± 65 |
| **Count of specific billing codes,^¶^** mean ± SD | 0.6 ± 0.8 | 0.9 ± 0.9 | 0.5 ± 0.8 |
| Alopecia | 2% (16) | 3% (5) | 0% |
| Arthritis | 23% (203) | 40% (61) | 21% (14) |
| Fatigue | 19% (169) | 25% (38) | 14% (9) |
| Interstitial Lung Disease | 2% (13) | 1% (1) | 2% (1) |
| Pulmonary Hypertension | 1% (9) | 1% (2) | 2% (1) |
| Rash | 9% (81) | 11% (16) | 9% (6) |
| Raynaud’s | 1% (12) | 5% (7) | 0% |
| Serositis | 4% (34) | 4% (6) | 8% (5) |
| Sicca | 0.3% (3) | 0% (0) | 0% (0) |

^*^Individuals with unclear diagnoses were individuals where the diagnosis was not confirmed by either an internal or external rheumatologist or in question by a rheumatologist.

‡Race, ethnicity, and lab values have missing data; see Supplemental Table 7 footnote. For unclear subjects, 7 (11%) for race, 11 (17%) for ethnicity, 11 (17%) for white blood cell count, 11 (17%) for platelet count, and 10 (15%) for serum creatine. ‡For ANA titer, up until July 1, 2016, titers were reported as 1:40 (negative), 1:80, and ≥ 1:160. After this date, titers were then reported as 1:40 (negative), 1:80, 1:160, 1:320, 1:640, 1:1280, and 1:2560.

§Presence of other autoantibodies included rheumatoid factor, cyclic citrullinated peptide, SSA (Ro), SSB (La), scl-70, centromere, RNP, Smith, dsDNA, ANCA, Jo-1, or any antibody from the myositis antibody panel.

^||^See Supplemental Table 4 for full list of ICD-9 and ICD-10-CM billing codes and Supplemental Table 5 for details on scoring. For each individual, we counted if any billing code was ever present (1 for present, 0 for absent) for each of the nine categories (i.e., arthritis, fatigue) and then summed this up across the nine prespecified billing code categories for a maximum score of nine.

**Supplemental Table 8. Alternative diagnoses for individuals with positive antinuclear antibodies who saw rheumatology in the training set.**

| **Diagnoses** | **N** |
| --- | --- |
| Fibromyalgia | 18 |
| Osteoarthritis | 11 |
| Gout | 6 |
| Hypermobility | 3 |
| Autoimmune thyroid disease | 3 |
| Inflammatory eye disease | 2 |
| Morphea | 2 |
| Primary Raynaud’s syndrome | 2 |
| Interstitial lung disease  Chronic pain | 2  2 |
| Cutaneous lupus | 1 |
| Aortitis | 1 |
| Pericarditis | 1 |
| Eosinophilia | 1 |
| Monoclonal gammopathy of unclear significance (MGUS) | 1 |
| Encephalitis | 1 |
| Jessner’s lymphocytic infiltrate (benign lymphocytic infiltration of skin)  Autoimmune hepatitis  Osteoporosis | 1  1  1 |

**
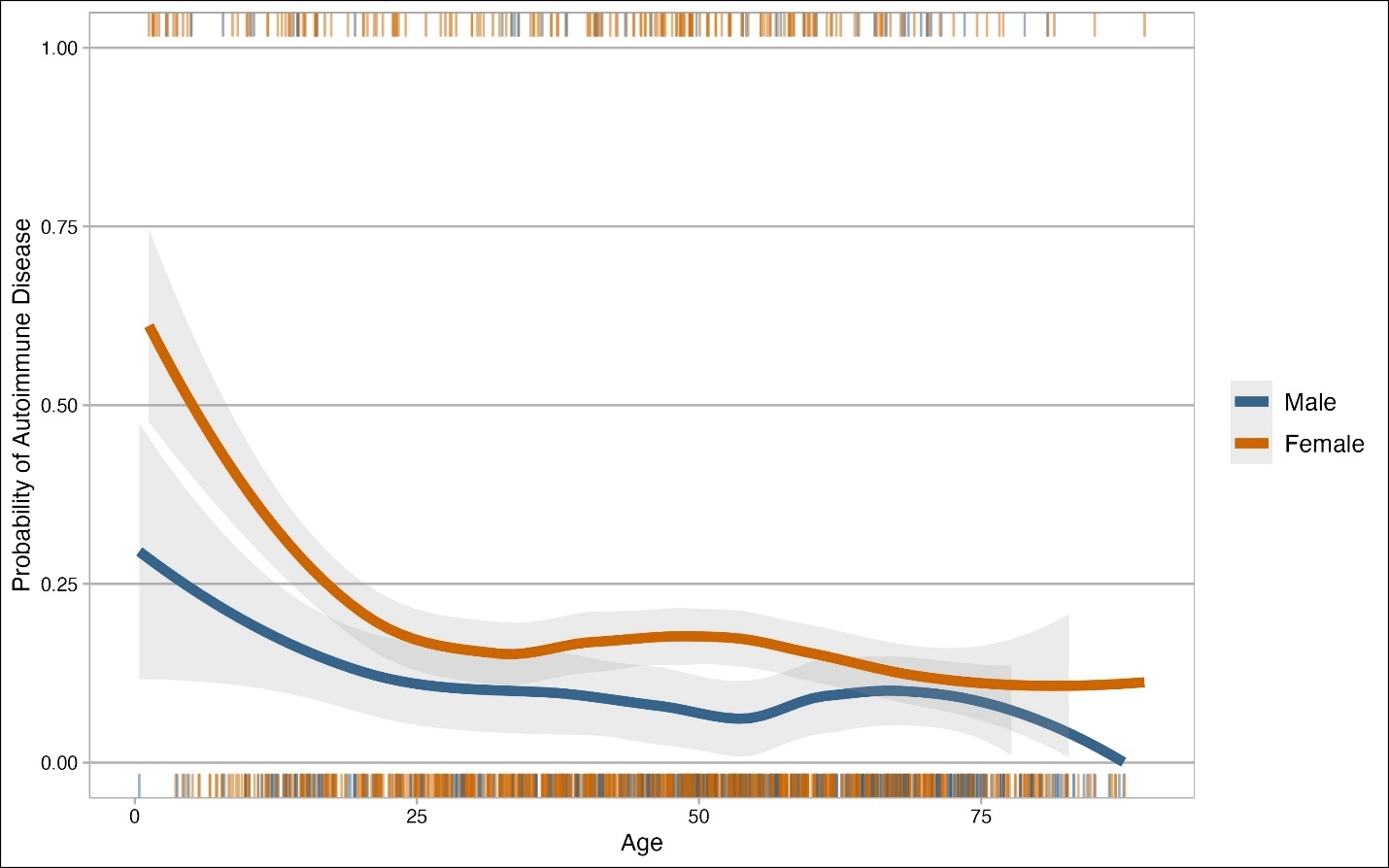
**

**Supplemental Figure 3. Probability of systemic autoimmune disease based on sex and age.** The y axis shows probability of systemic autoimmune disease and x axis age at positive ANA. The blue line indicates male individuals, and the orange line indicates female individuals with the shadowing around the line showing 95% confidence intervals. The rug plot shows individual data points for individuals with autoimmune disease on the top and individuals without autoimmune disease on the bottom.

**Supplemental Table 9. Characteristics of positive ANA individuals with vs. without systemic autoimmune disease in the validation set.**

| **Characteristics** | **No systemic autoimmune disease**  **n = 375** | **Systemic autoimmune disease**  **n = 74** | ***p* value^*^** |
| --- | --- | --- | --- |
| **Age at positive ANA**, years, mean ± SD | 49.5 ± 19.7 | 40.1 ± 21.6 | < 0.001 |
| **Race % (n)**^†^  White | 85% (294) | 86% (61) | 0.62 |
| African American | 12% (42) | 12% (8) |  |
| Asian | 1% (5) | 0% (0) |  |
| Native American | 1% (1) | 1% (1) |  |
| Other | 1% (4) | 1% (1) |  |
| **Ethnicity**^†^ |  |  | 0.46 |
| Hispanic | 3% (10) | 1% (1) |  |
| Not Hispanic or Latino | 97% (327) | 99% (70) |  |
| **Gender** |  |  |  |
| Female | 73% (273) | 81% (60) | 0.14 |
| Male | 27% (102) | 19% (14) |  |
| **ANA titer**^‡^  1:80 | 20% (76) | 15% (11) | 0.28 |
| ≥ 1:160 | 80% (299) | 85% (63) |  |
| **White blood cell count**^†^  K/uL, Mean ± SD | 6.9 ± 2.9 | 7.0 ± 2.4 | 0.38 |
| **Platelet count**^†^  K/uL, Mean ± SD | 224 ± 88 | 275 ± 100 | <0.001 |
| **Serum creatinine**^†^  mg/dL, Mean ± SD | 1.3 ± 1.5 | 0.9 ± 0.4 | 0.003 |
| **Ever present autoantibody**^§^  No | 90% (337) | 59% (44) | <0.001 |
| Yes | 10% (38) | 41% (30) |  |
| **Total any billing codes**, mean ± SD | 38 ± 71 | 31 ± 66 | 0.74 |
| **Count of specific billing codes**, ^\|\|^ mean ± SD | 0.7 ± 0.9 | 1.0 ± 0.8 | 0.01 |
| Alopecia | 1% (4) | 3% (2) | 0.26 |
| Arthritis | 28% (104) | 49% (36) | <0.001 |
| Fatigue | 22% (82) | 30% (22) | 0.14 |
| Interstitial Lung Disease | 3% (10) | 1% (1) | 0.50 |
| Pulmonary Hypertension | 2% (6) | 0% | 0.27 |
| Rash | 10% (36) | 8% (6) | 0.69 |
| Raynaud’s | 3% (10) | 3% (2) | 0.99 |
| Serositis | 6% (21) | 3% (2) | 0.30 |
| Sicca | 1% (5) | 0% | 0.32 |

^*^Mann-Whitney U test for continuous variables and chi-square test for categorical variables. P values calculated with excluding missing observations.

†Race, ethnicity, and lab values have missing data with 32 (7%) for race, 41 (9%) for ethnicity, 91 (20%) for white blood cell count, 95 (21%) for platelet count, and 100 (22%) for serum creatine.

^‡^For ANA titer, up until July 1, 2016, titers were reported as 1:40 (negative), 1:80, and ≥ 1:160. After this date, titers were then reported as 1:40 (negative), 1:80, 1:160, 1:320, 1:640, 1:1280, and 1:2560.

^§^Presence of other autoantibodies included rheumatoid factor, cyclic citrullinated peptide, SSA (Ro), SSB (La), scl-70, centromere, RNP, Smith, dsDNA, ANCA, Jo-1, or any antibody from the myositis antibody panel.

^||^See Supplemental Table 4 for full list of ICD-9 and ICD-10-CM billing codes and Supplemental Table 5 for details on scoring. For each individual, we counted if any billing code was ever present (1 for present, 0 for absent) for each of the nine categories (i.e., arthritis, fatigue) and then summed this up across the nine prespecified billing code categories for a maximum score of nine.


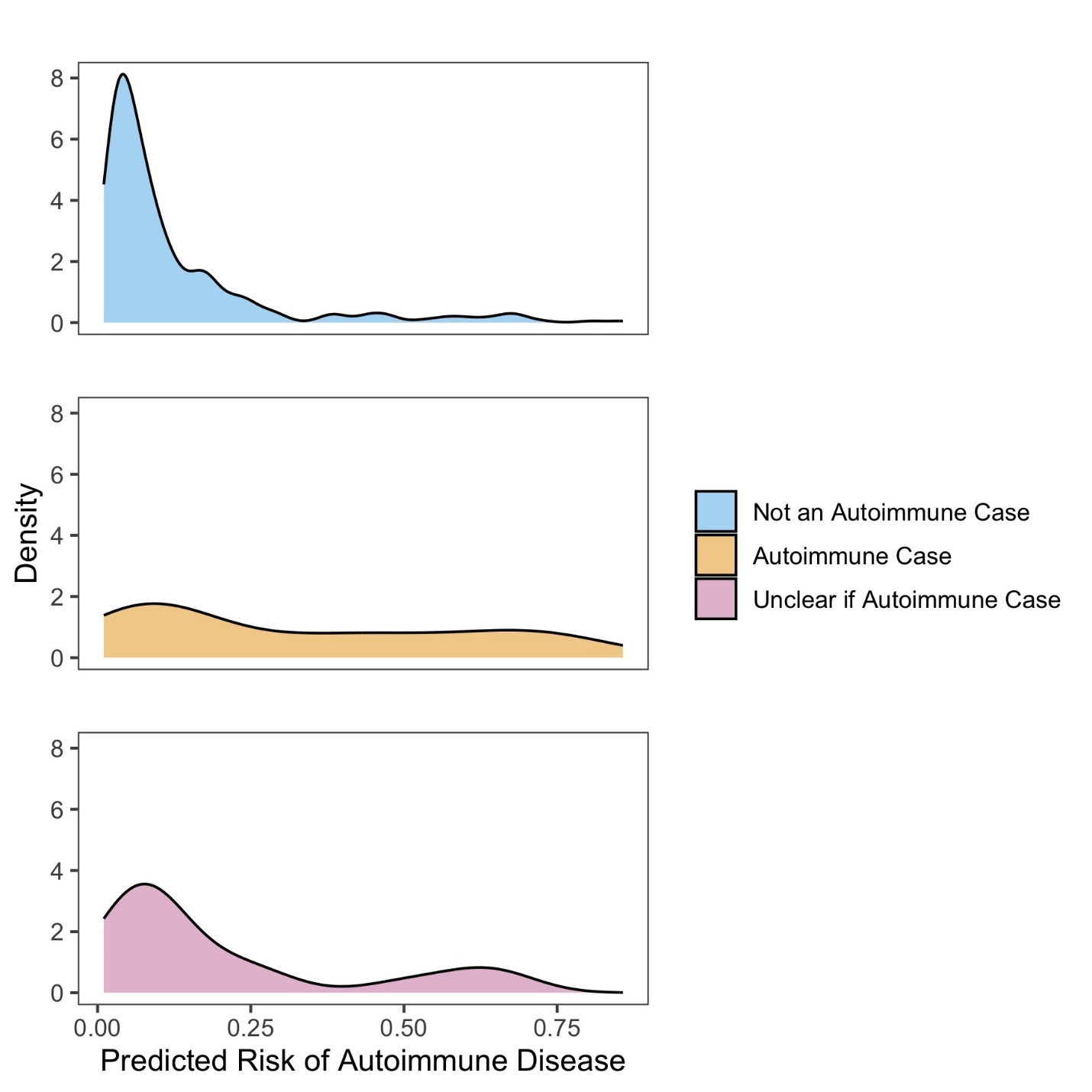


**Supplemental Figure 4. Distribution of autoimmune disease risk scores by case status.** The density plot shows the distribution of risk scores for individuals who are not cases (blue), individuals who are cases with autoimmune diseases (orange), and unclear autoimmune disease status (pink). The x axis shows risk scores and y axis density or number of individuals.

**
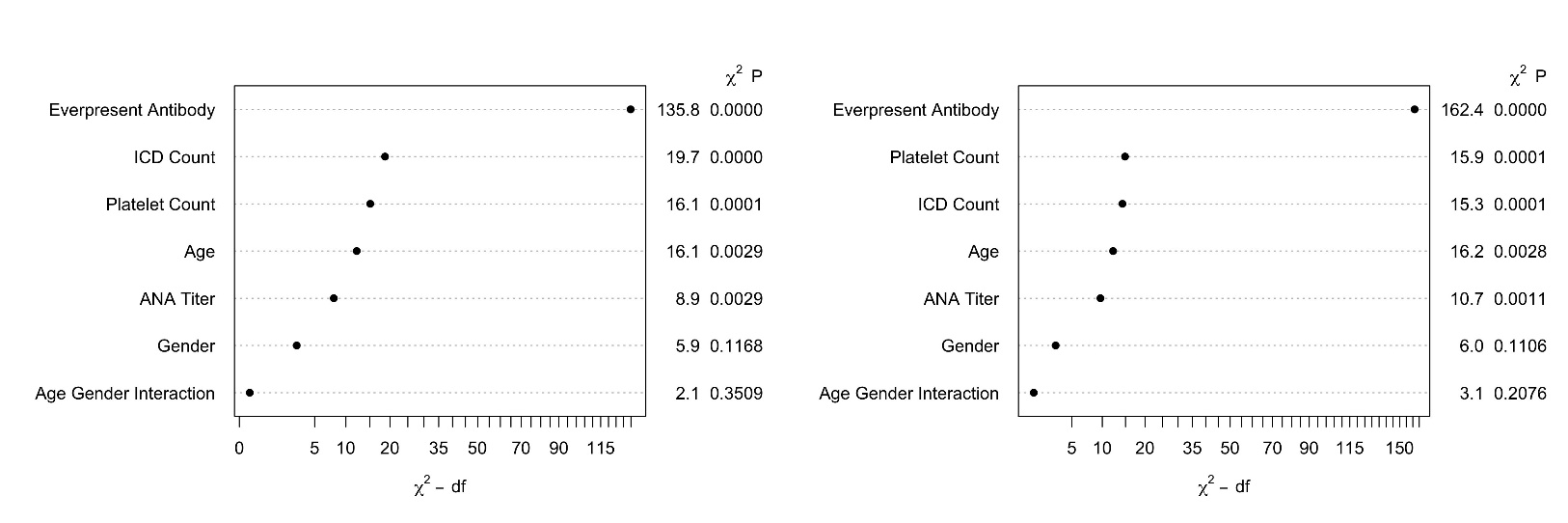
**

**Supplemental Figure 5. Comparison of importance of variables in logistic regression and Cox models**. **(A)** shows the list of variables for the logistic regression model to the left with p values to the right. The x axis shows variable importance using a Wald statistic. **(B)** shows the list of variables for the Cox model.

Supplemental Table **10**. ANA titer by case status.

| **ANA titer^*^** | **Autoimmune disease**  **% (n)**  **n = 96** | **No autoimmune disease**  **% (n)**  **n = 488** |
| --- | --- | --- |
| 1:80 | 4% (4) | 7% (33) |
| 1:160 | 29% (28) | 39% (190) |
| 1:320 | 27% (26) | 36% (178) |
| 1:640 | 6% (6) | 12% (59) |
| 1:1280 | 25% (24) | 4% (21) |
| ≥ 1:2560 | 9% (8) | 1% (7) |

^*^Positive ANA individuals come from a cohort from 2017-2021 where granular ANA titer reporting was available.

**
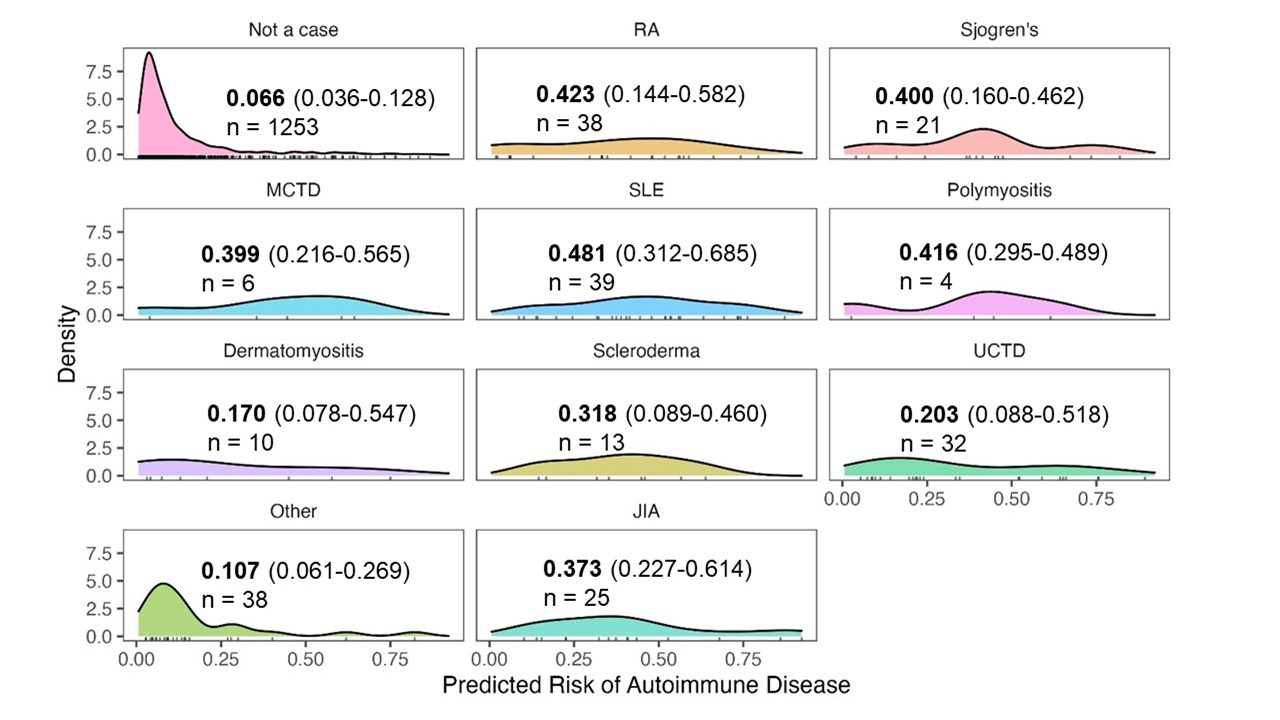
**

**Supplemental Figure 6. Distribution of risk scores by autoimmune disease type.** Data is from a de-identified EHR database that includes training and validation sets. Different colors representing different autoimmune diseases are shown with the disease label in each box. For each autoimmune disease type including subjects without autoimmune disease or not a case, distribution of risk scores with a rug plot on the x axis are shown and density on y axis. Bolded numbers represent median risk score with interquartile ranges in parentheses. RA = rheumatoid arthritis, MCTD = mixed connective tissue disease, SLE = systemic lupus erythematosus, UCTD = undifferentiated connective tissue disease, Other represents seronegative conditions such as plaque psoriasis, psoriatic arthritis, and inflammatory bowel disease, JIA = juvenile idiopathic arthritis.

**
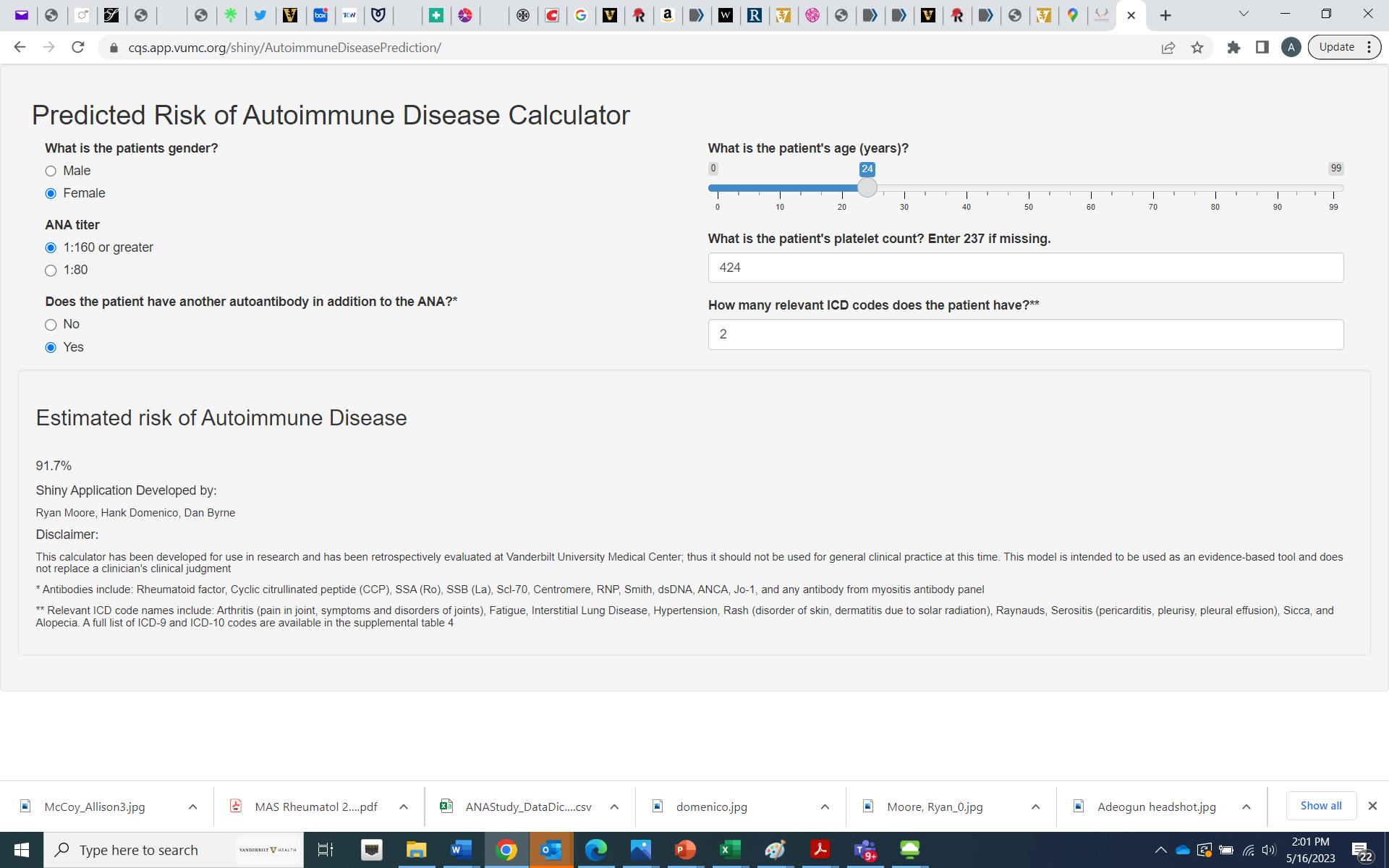
**

**Supplemental Figure 7. Screenshot of Shiny app for risk model for systemic autoimmune disease.** The screenshot shows the risk model covariates used to estimate risk for systemic autoimmune disease. The Shiny app can be accessed at the following link: https://cqs.app.vumc.org/shiny/AutoimmuneDiseasePrediction/

**Appendix Text**

**ANA Reporting, Titers, and Autoantibodies**

For the ANA screen, the ANA Hep-2 IFA assay was used for the entire study period. Before 2014, the assay was performed and read manually. After 2014, the assay was moved to the QuantaLyser 240, which allowed automatic staining and interpretation.

For ANA titers, up until July 1, 2016, titers were reported as either 1:80 or ≥ 1:160. After this date, positive ANA titers were reported as 1:80, 1:160, 1:320, 1:640, 1:1280, and 1:2560. Primary analysis treated ANA titer as a binary outcome with 2 categories 1:80 and ≥ 1:160 with 1:160, 1:320, 1:640, 1:1280, and 1:2560 from the recent ANA reporting collapsed into the ≥ 1:160 category. Sensitivity analyses were conducted using data from 2017 to 2021 where the full spectrum of ANA titers (1:80, 1:160, 1:320, 1:640, 1:1280, and 1:256) were used in the model.

All autoantibodies were measured via enzyme-linked immunosorbent assays with manufacturer values to determine positivity. For the ENA assays, the Zeus ENA profile-6 test system was used for SSA, SSB, Smith, and RNP antibodies, the Inova Scl-70 assay for Scl-70 antibodies, and Bio-Rad Anti-dsDNA assay for dsDNA. These tests are all performed on the Dynex Agility instrument. The immunoassay for SSA captures both Ro60 and Ro52.

**Machine Learning Methods**

Extreme gradient boosting (XGB) is a machine learning method that is the summation of multiple models where each successive model attempts to correct errors in the previous model to improve overall performance. We fit an XGB model with a max tree depth of 5 and 5 boosting iterations using the R package (4.2.1) xgboost. Neural networks represent a subset of machine learning methods that can handle complex data and interactions. We built a neural network with four hidden layers using the the R package neuralnet.

**Sensitivity Analyses – Cox Model**

To account for longitudinal and censored data, we conducted a Cox proportional-hazard model using the same variables as those in the logistic regression model. We assessed if model predictors behaved similarly in predicting the binary outcome of presence of systemic autoimmune disease for the logistic regression model compared to time to development of systemic autoimmune disease for the Cox model.

**Sensitivity Analyses – Case definition**

Primary analysis included all systemic autoimmune diseases listed in Supplemental Table 1. As the goal of the risk model was to identify individuals with systemic autoimmune diseases that would follow in the rheumatology clinic, we included seronegative conditions (i.e. psoriatic arthritis, ankylosing spondylitis). For a sensitivity analysis, individuals with seronegative conditions were not counted as cases.

**Model Validation**

For the bootstrap validation using 200 replications, we observed minimal optimism in c-statistic (0.829 in original cohort compared to 0.817 optimism corrected), Brier score (0.095 in original cohort compared to 0.097 optimism corrected), calibration slope (1.0 in original cohort compared to 0.950 optimism corrected), and calibration intercept (0.0 in original cohort compared to -0.06 optimism corrected).
